# Highly Sensitive Strain Sensor Based on a Novel Mach-Zehnder Interferometer with TCF-PCF Structure

**DOI:** 10.3390/s18010278

**Published:** 2018-01-18

**Authors:** Xinran Dong, Haifeng Du, Zhi Luo, Ji’an Duan

**Affiliations:** State Key Laboratory of High Performance Complex Manufacturing, College of Mechanical and Electrical Engineering, Central South University, 932 South Lushan Street, Changsha 410083, China; xrdong@csu.edu.cn (X.D.); duhaifeng@csu.edu.cn (H.D.)

**Keywords:** Mach-Zehnder interferometer (MZI), photonic crystal fiber (PCF), thin core fiber (TCF), strain sensor

## Abstract

A highly sensitive strain sensor based on a novel fiber in line Mach-Zehnder interferometer (MZI) was demonstrated experimentally. The MZI was realized by splicing a section of photonic crystal fiber (PCF) with the same length of thin core fiber (TCF) between two single mode fibers (SMFs). The fringe visibility of MZI can reach as high as 20 dB in air. In particular, the strain sensitivity of −1.95 pm/με was achieved within a range from 0 to 4000 με. Furthermore, the strain properties of different length of MZI was investigated. It was found that the sensitivity was weekly dependent on the length of MZI. The strain sensitivities corresponding to the MZI with 35 mm PCF, 40 mm PCF and 45 mm PCF at 1550 nm band were −1.78 pm/με, −1.73 pm/με and −1.63 pm/με, respectively. Additionally, the sensor has advantages of simple fabrication, compact size and high sensitivity as well as good fringe visibility.

## 1. Introduction

Optical fiber sensors has been widely used to monitor some parameters in sensing fields, such as temperature [1], refractive index [2], magnetic field [3], humidity [4], strain [5], which is due to their unique advantages of compact size, high resolution and immunity to electromagnetic interference [6,7]. Among them, strain has played a critical role in the application of health inspection of bridges and buildings. Especially, the PCFs combined with functional materials are popular for sensing application at present and attract lots of attention [8,9]. Various strain sensors based on fiber Bragg gratings [10], long period fiber gratings [11], Fabry-Perot interferometer [12], Sagnac loop [13], Mach-Zehnder interferometer [14,15] and photonic crystal fiber (PCF) interferometer [16,17,18,19,20,21] have been proposed and demonstrated. Among them, PCF-based MZIs have been proved to own high strain sensitivity while low temperature sensitivity, which reduces the effect of temperature cross sensitivity. For instance, in 2007, Dong [17] reported a temperature-insensitivity strain sensor based on polarization-maintaining PCF (PM-PCF) interferometer. The measurement range was up to 32 mε. However, the sensitivity was only 0.23 pm/με. In 2008, Choi [22] reported a new PCF-MZI structure with a high sensitivity of −1.8 pm/με within range from 0 to 2200 με. The structure was implemented by fabricating an LPFG in PCF and splicing an air hole collapsing in other end of fiber, which was too complex and time-consuming to fabricate a LPFG in PCF. In 2013, Zheng [23] demonstrated a strain sensor with a sensitivity as high as 2.1 pm/με based on normal PCF-MZI, the MZI was induced by core mode and the high order core mode rather than the cladding mode because of the special air hole array of the PCF. Meanwhile, the fringe visibility was about 12 dB. In 2016, Dash [20] fabricated a MZI based on tapered PCF with an up-tapered joint. The measurement sensitivity was 1.6 pm/με, however, the tapered collapsed region could reduce the mechanical strength and cause larger lost. In 2017, Villatoro [24] proposed a novel strain sensor based on multi-core fiber (MCF) interference. The strain sensitivity was about 1.7 pm/με, which was slightly smaller than that of our proposed MZI, but it has a big advantage of its capability to high temperatures up to 1000 °C as well as larger measurement range up to 3800 με. In addition, it is worth mentioning that the polymer fibers are more suitable for strain sensing applications because of the larger elasticity than silica fiber and lower Young’s modulus [25,26,27]. At the same time, different polymer fibers with polycarbonate [25] and Zeonex-PMMA [28] have been reported. For example, in 2017, Oliveira [26] has reported a sensor with a high strain sensitivity of 3.03 pm/με in the range of 0~15,000 με and a temperature sensitivity of 103.6 pm/°C in the range of 20 °C~100 °C, which was a SMS structures based on a multimode polymer optical fiber.

In this paper, we proposed a novel strain sensor based on MZI with TCF-PCF structure, which was formed by splicing a piece of TCF with the same length of PCF between two SMFs. The fusion point was more robot than that of up-tapered joint or collapsed fusion region, which making the sensor having merits of good mechanical strength and low fusion lost. In addition, a high strain sensitivity of −1.95 pm/με with a good linearity of 0.9971 in the range of 0~4 mε was achieved. Moreover, the dependence of the stain sensitivity on the length of MZI was also investigated experimentally.

## 2. Fabrication of the Sensor and Sensing Principle

### 2.1. Fabrication of the Sensor

**Figure 1:**
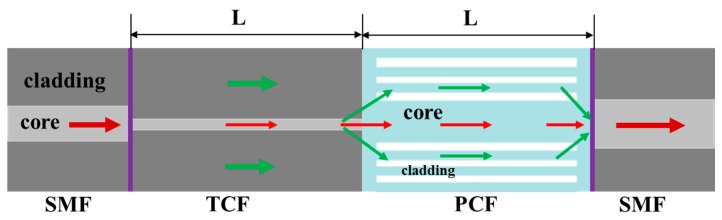
Schematic diagram of the MZI based on TCF-PCF structure.

**Figure 2:**
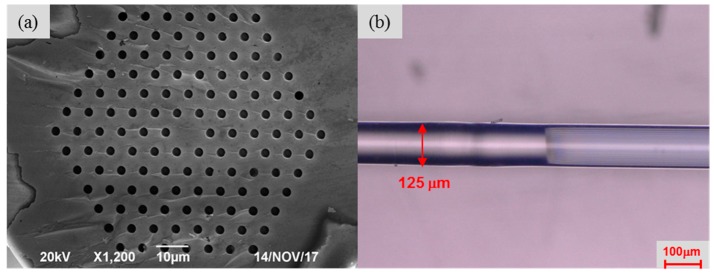
(**a**) SEM image of the cross section of PCF (**b**) microscope image of the fusion of TCF and PCF.

Figure 1 shows the schematic diagram of the MZI based on TCF-PCF composite structure. A short piece of TCF (460-HP, Nufern, East Granby, CT, USA) and the same length of PCF (LMA-10, NKT Photonics, Blokken, Denmark) was spliced between two SMFs (SMF-28e, Corning, Shanghai, China) by using a fiber cleaver and a fusion splicer (FSM 80 s, Fujikura, Tokyo, Japan). The fibers were all removed the coating layer before splicing. The core and cladding diameter of TCF employed in experiment were 2.5 μm and 125 μm, respectively. The choice of TCF was due to its smaller core diameter than that of SMF and the different elastic coefficient with PCF. On the one hand, the mode field diameter of TCF was much smaller, the evanescent wave of the fundamental of the fundamental guide mode could be enhanced, leading to the light injected to the cladding mode of PCF effectively [29,30]. On the other hand, if we assumed that the fiber structure was elastic bod, when the same strain was applied along the fiber, the stress of the fiber cross section of the TCF-PCF structure MZI would be larger than that of single PCF or TCF structure MZI, according to Hooke’s law. This could be increased the photo-elastic effect in fiber, leading to the strain sensitivity enhancement. The SEM of cross section of the PCF used is shown in Figure 2a. The PCF consists of six layers of air holes and it had a core diameter of 10.1 μm and cladding diameter of 125 μm. Figure 2b shows the fusion region of TCF and PCF. The schematic of the sensor is set up in Figure 1. When the light passes through SMF into the TCF, more power are injected into the cladding mode of TCF due to its smaller diameter of fiber core. At the TCF-PCF fusion point, the fundamental mode of TCF will begin to diffract, a part of the power is coupled into the cladding modes of the PCF and some are transmitted in fiber core at the same time. And then they reach the spliced point of SMF-PCF, light in fiber cladding of PCF will be coupled back to fiber core and interference with light in fiber core. This structure was robust than that of MZI with up-tapered joints [20] and S-tapered structure [31] as well as MZI with rectangular air bubble [32]. Furthermore, in order to trace the transmission spectrum change, a broadband ASE source (C+L, 1528 nm~1602 nm) and an optical spectrum analyzer (OSA) with a resolution of 0.01 nm (OSA, Agilent 86142B, Agilent Technologies, Santa Clara, CA, USA, 600 nm~1700 nm) were connected to the two ends during the fabrication processing.

### 2.2. Sensing Principle

As shown in Figure 1, the input light would be transmitted in the fiber core and fiber cladding, and the two paths of light in fiber core and cladding will produce interference at the fiber end. For the MZI, the m order cladding modes resonant wavelength can be simply written as [23]:

(1) $$\lambda_{m}=\frac{\Delta n_{eff}L}{2m+1}$$

where $\Delta n_{eff}=n_{eff}^{core}-n_{eff}^{cl,m},\;n_{eff}^{core}$ and $n_{eff}^{cl,m}$ are the refractive indices of fundamental mode and m order cladding modes, respectively. $\lambda_{m}$ is the resonant wavelength, L is the physical length of the MZI.

When axial strain is applied, the length of MZI will be increased, the resonant wavelength shift can be expressed by differentiating the Equation (1), which can be given as [16,23]:

(2) $$\Delta \lambda_{m}=\left[1+\left(\frac{L}{\Delta n_{eff}}\right)\cdot \left(\frac{\partial \left(\Delta n_{eff}\right)}{\partial L}\right)\right]\cdot \lambda_{m}\varepsilon$$

It can be seen that the resonant wavelength shift is a linear function of applied strain ε. Meanwhile, the strain sensitivity is mainly dependent on the change of $\Delta n_{eff}$ induced by the extended length of MZI, that is, $\partial \left(\Delta n_{eff}\right)/\partial L$. Additionally, the strain would also produce a physical deformation of the splicing joints. Therefore, the output light intensity would be change as the strain increases.

## 3. Experimental Results and Discussion 

**Figure 3:**
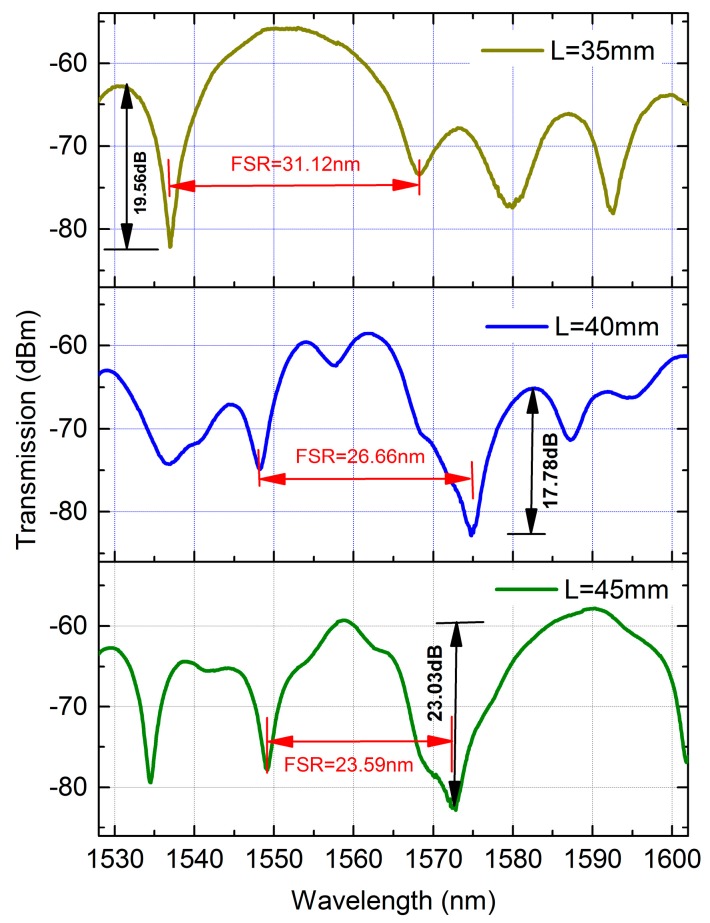
Transmission spectra of the proposed MZI with different length of PCF.

The transmission spectra of the TCF-PCF structure with 35 mm, 40 mm and 45 mm length of PCF are shown in Figure 3. It can be seen the proposed MZI has exhibited good spectrum properties. The fringe visibility of the MZI with 35 mm PCF, 40 mm PCF and 45 mm PCF were as high as 19.56 dB, 17.78 dB and 23.03 dB, respectively. Those were larger than that of two-taper MZI (17.5 dB) [33], taper-core-offset section MZI (11.3 dB) [34], step-like taper MZI (18.64 dB) [35], normal PCF-MZI (14 dB) [23]. In addition, the free spectrum range (FSR) would be reduced as the length of MZI increased. The FSR of the MZI with 35 mm PCF, 40 mm PCF and 45 mm PCF were 31.12 nm, 26.66 nm and 23.59 nm, respectively, as shown in Figure 3. Additionally, we can see that the insertion loss of the proposed MZI is larger than that of MMF-TF structure MZI [30], which is due to the presence of air holes in the cladding of PCF, making the collapsing region larger. In order to reduce the insertion loss, the fusion parameters including arc power and arc time could be further optimized.

**Figure 4:**
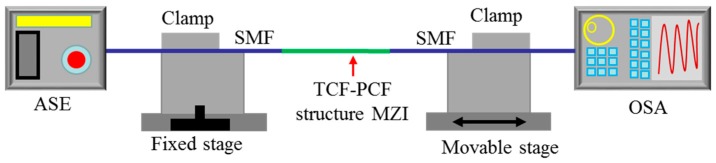
Experimental step for strain measurement.

Figure 4 shows the experimental step for strain measurement. The TCF-PCF structure MZI was fixed on two stages. One was fixed stage and the other was movable stage. Meanwhile, the ASE source and OSA were connected to monitor the transmission spectra change in real time.

**Figure 5:**
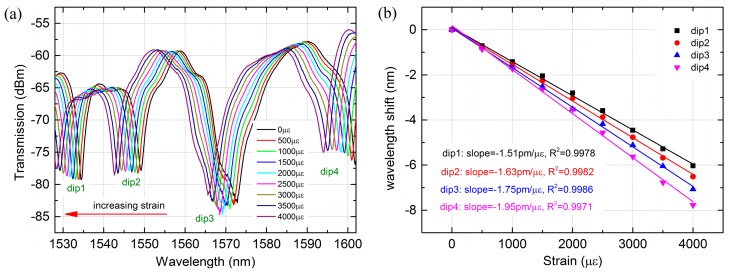
(**a**) Transmission spectra change of the MZI with 45 mm PCF as the strain increases; (**b**) wavelength shifts of three dips as a function of strain.

Figure 5 shows the transmission spectra change of MZI with 45 mm PCF as the applied strain increases. It can be seen that the whole resonant wavelength shifted towards shorter wavelength direction when the strain was increased gradually from 0 to 4000 με. The total wavelength shift variation of the dip1, dip2, dip3 and dip4 were 6.03 nm, 6.51 nm, 7.07 nm and 7.77 nm, respectively, as shown in Figure 5a. A linear curve fitting of the wavelength shift and strain is illustrated in Figure 5b. The measure strain sensitivities for the four dips were −1.51 pm/με, −1.63 pm/με, −1.75 pm/με and −1.95 pm/με, respectively. Meanwhile, the linearity of wavelength shift to strain response was excellent. A high R^2^ of 0.9978, 0.9982, 0.9986 and 0.9971 for dip1, dip2, dip3 and dip4 were obtained, respectively. From the investigation, it is found that the strain sensitivity of the MZI is closed to the resonant wavelength of cladding modes and the wavelength with higher order cladding modes tend to be more sensitive to the applied strain. This is because the $\Delta \lambda_{m}$ will vary when $\Delta n_{eff}$ change according to Equation (2).

**Figure 6:**
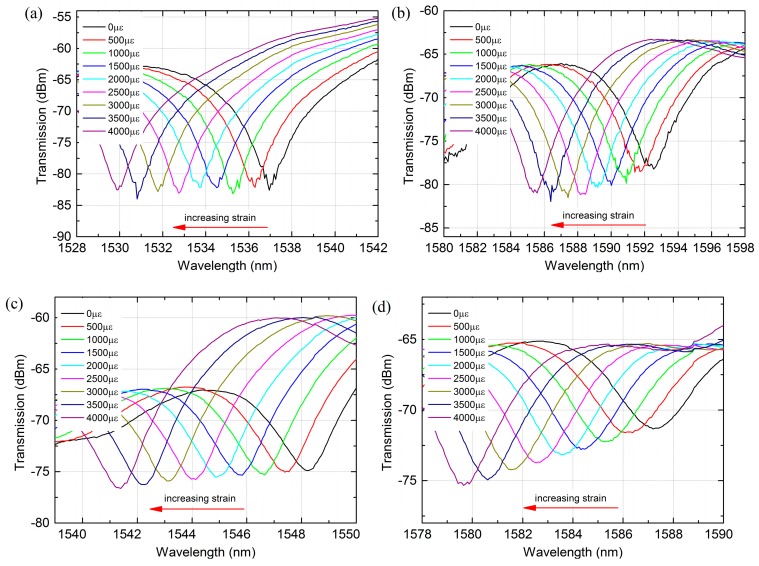
Transmission spectra change as the strain increases: (**a**) 1536 nm dip of the MZI with 35 mm PCF; (**b**) 1592 nm dip of the MZI with 35 mm PCF; (**c**) 1548 nm dip of the MZI with 40 mm PCF; (**d**) 1587 nm dip of the MZI with 40 mm PCF.

**Figure 7:**
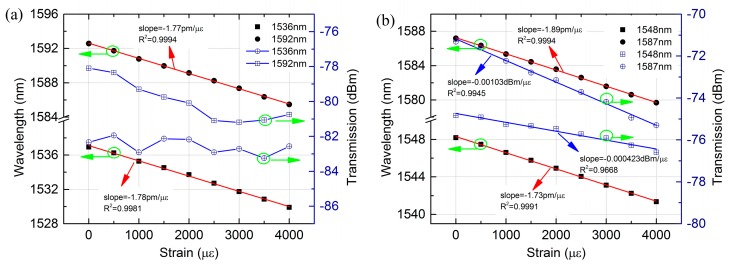
Wavelength shifts and transmission loss of different dips as a function of strain: (**a**) the MZI with 35 mm PCF; (**b**) the MZI with 40 mm PCF.

Additionally, the strain characteristics of sensor with different length were also investigated experimentally. In order to compare the strain characteristics of low order cladding mode and high order cladding modes, we chosen two dips around 1550 nm and 1580 nm to compare and analysis. From Figure 6a,b, it was observed that the 1536 nm and 1592 nm dip of the MZI with 35 mm PCF experienced a blue-shift with 7.04 nm and 7.1 nm, respectively, when the strain increased from 0 to 4000 με. For the MZI with 40 mm PCF as shown in Figure 6c,d, the wavelength shift variations of 1548 nm and 1587 nm dip were 6.84 nm and 7.5 nm at the same strain range. By contrast, we can see that the resonant wavelength with higher order cladding mode has exhibited slightly sensitive to the applied strain and the wavelength shift and strain was perfect with linear fitting with an excellent linearity of above 0.999, which is shown in Figure 7. The strain sensitivity of 1536 nm and 1592 nm dip were −1.78 pm/με and −1.77 pm/με, respectively, by linear fitting, as shown in Figure 7a. Meanwhile, a strain sensitivity of −1.73 pm/με and −1.89 pm/με for 1548 nm and 1587 nm dip were achieved by linear fitting, respectively, as shown in Figure 7b. From the investigation, it is found that the strain sensitivity of MZI with 40 mm PCF is slightly larger than that with 35 mm PCF. However, the strain sensitivity is weekly dependent on the length of PCF, as exhibited in Figure 5b and Figure 7. The sensitivities we obtained were considerably higher than that of long period fiber gratings fabricated by CO_2_ laser (0.63 pm/με) [36], Bragg gratings (1.2 pm/με) [31] and some strain sensors based on PCF-MZIs. For instances, cascaded PCF-MZI (1.07 pm/με) [37], polarization-maintaining photonic crystal fiber (PM-PCF) based Sagnac interferometer (0.23 pm/με) [17], twin-core photonic crystal fiber (TC-PCF) interferometer (−0.31 pm/με) [19], tapered PCF with up-tapered joint (−1.6 pm/με) [20], PM-PCF (∼1.01 pm/με) [38] and PCF with two asymmetric cores (−1.22 pm/με) [5]. In addition, the sensitivity we reported was smaller than that of S-tapered PCF-MZI (4.3 pm/με) [31] and modified PCF-MZI (−1.98 pm/με) [18]. However, the strain sensor we proposed has the merits in simple fabrication and larger strain measurement range as well as better fringe visibility.

The transmission loss change of the MZI with different length of PCF was also illustrated in Figure 7. The transmission loss increased gradually as the applied strain increased as shown in Figure 6. For the MZI with 35 mm PCF as shown in Figure 7a, the transmission loss change was nonlinear as the strain increased. The transmission loss of 1592 nm dip decreased from −79.38 dBm to −77.47 dBm whereas that of 1536 nm dip was almost unchanged when the strain was increased from 0 to 4000 με. However, that of MZI with 40 mm PCF was perfect with linear fitting. The fitting slope of 1548 nm and 1587 nm dip were −4.23 × 10^−4^ dBm/με with a linearity of 0.9668 and −10.3 × 10^−4^ dBm/με with a linearity of 0.9945, respectively. From the results, it can be seen that the transmission loss change depends on the cladding mode and the length of MZI.

## 4. Conclusions

In conclusion, we have achieved a strain sensor based on TCF-PCF structure MZI, which is fabricated by splicing a section of TCF and PCF between two SMFs. The novel structure MZI has shown better fringe visibility than that of normal PCF-MZI and a strain sensitivity as high as −1.95 pm/με was achieved in the range of 0~4000 με, which is larger than that of MZI based on tapered PCF and PM-PCF. In addition, the proposed sensor is easy to fabricate and exhibits large strain measurement range as well as low production lost, which means that the structure is attractive for the development of strain sensors.
